# Risks of Intimate Partner Violence for Women Living with HIV Receiving Cash Transfers: A Qualitative Study in Shinyanga, Tanzania

**DOI:** 10.1007/s10461-023-03997-2

**Published:** 2023-01-24

**Authors:** Rebecca Hémono, Agatha Mnyippembe, Atuganile Kalinjila, Jesca Msoma, Ndola Prata, William H. Dow, Claire Snell-Rood, Amon Sabasaba, Prosper Njau, Sandra I. McCoy

**Affiliations:** 1 Division of Epidemiology, School of Public Health, University of California, Berkeley, 2121 Berkeley Way, 94704 Berkeley, CA USA; 2 Health for a Prosperous Nation, Dar es Salaam, Tanzania; 3 The United Republic of Tanzania Ministry of Health, Dodoma, Tanzania

**Keywords:** Behavioral economics, Cash transfers, HIV, Intimate partner violence, Gender-based violence

## Abstract

Cash transfers are increasingly used to motivate adherence to HIV care. However, evidence on cash transfers and intimate partner violence (IPV) is mixed and little is known about their safety for women living with HIV. We conducted in-depth interviews with women living with HIV who participated in a randomized trial providing 6 months of cash transfers (~$4.5 or $11 USD) conditional on HIV clinic attendance in Shinyanga, Tanzania to assess how receiving cash affects IPV and relationship dynamics. Eligible participants were 18–49 years, received cash transfers, and in a partnership at baseline. Data were analyzed in Dedoose using a combined inductive-deductive coding approach. 25 interviews were conducted between November 2019-February 2020. Women’s employment was found to be a source of household tension and violence. None of the participants reported physical or sexual IPV in relation to cash transfers, however, some women experienced controlling behaviors or emotional violence including accusations and withholding of money, particularly those who were unemployed. Cash transfers were predominantly used for small household expenses and were not viewed as being substantial enough to shift the financial dynamic or balance of power within relationships. Our findings suggest that small, short-term cash transfers do not increase physical or sexual IPV for women living with HIV however can exacerbate controlling behaviors or emotional violence. Modest incentives used as a behavioral nudge to improve health outcomes may affect women differently than employment or larger cash transfers. Nonetheless, consultations with beneficiaries should be prioritized to protect women from potential IPV risks.

## Introduction

Economic interventions including cash transfers have been used to address poor engagement and retention in HIV care and support the UNAIDS 95-95-95 targets [1]. A growing number of studies suggest that cash transfers can improve adherence to antiretroviral therapy (ART) through motivating behavior change and addressing poverty as a structural barrier to care [2–6]. These findings, together with UNAIDS support for cash transfers, have led to increased investment of this approach for people living with HIV [1]. However, evidence on the effects of cash transfers on intimate partner violence (IPV) is mixed.

Cash transfers are commonly used as a strategy to combat IPV, hypothesized to reduce poverty-related household stress and improve women’s bargaining power in relationships [7–9] Some studies have demonstrated that offering households cash can decrease IPV [8, 10–14], while others have reported heterogeneous effects, including exacerbated IPV risks in the presence of cash transfers [13, 15–17]. The influence of cash transfers on IPV has also been shown to vary with different delivery mechanisms, cash amounts, and beneficiary populations. For example, a study in Ecuador found both increases and decreases in emotional IPV among cash transfer beneficiaries depending on the couple’s level of education [8]. In the *Oportunidades* program, the smaller cash transfer group experienced decreased IPV while the larger transfers were associated with increased aggressive behaviors [15]. Effects of *Oportunidades* were also inconsistent across different forms of violence, with physical violence decreasing by 40% and emotional violence and threats increasing moderately among beneficiaries [13.

In addition to the heterogeneity in the existing literature, there is a paucity of evidence examining how cash transfers affect IPV among women living with HIV. IPV is pervasive among women living with HIV in sub-Saharan Africa [18], who systematically experience higher odds of violence compared to the general population [19–21]. Women in violent partnerships confront additional barriers to initiating and adhering to ART due to verbal threats, intimidation, mistrust, and lack of financial independence from male partners [22–27]. These forms of passive and active interference increase risk of ART disengagement and contribute to onward viral transmission [22–25].

Given the mixed findings on cash transfers and IPV in the general population and the scale and scope of interventions under consideration to improve HIV outcomes, it is critical to determine whether this approach is safe and beneficial for women living with HIV, who comprise a large population. Most prior studies have investigated cash transfer safety using quantitative approaches and there is limited understanding of how women perceive their risk of IPV to be influenced when receiving financial incentives. To address this gap, we conducted a qualitative study with women living with HIV who participated in a cash transfer trial in Shinyanga Region, Tanzania to explore beneficiary perspectives on cash transfers. The main objective of our study was to understand how cash transfers affect relationship safety and IPV. We also aimed to understand the extent to which women experience their partners interfering with their access and ability to remain in HIV care.

## Methods

### Study Population and Setting

This qualitative study was conducted with participants from a three-arm randomized trial investigating the effect of cash transfers on viral suppression (NCT03351556) [3, 28] in Shinyanga, Tanzania, a resource-constrained region in the Lake Zone with an HIV prevalence of 6% among adults aged 15 and older [29] and an IPV prevalence of 78% among ever-married women between 15 and 49 years [30]. Study sites included two large clinics, a per-urban clinic, and a dispensary.

Participants in the trial were enrolled from April-December 2018 and randomized in a 1:1:1 ratio to receive six months of cash transfers of 10,000 TZS (~ 4.5 USD) or 22,500 TZS (~ 11 USD) per month via mobile money, conditional on HIV clinic attendance, or standard HIV services (n = 530). The goal of the cash transfer was to motivate engagement in HIV care, with cash amounts designed to subsidize the cost of food, clinic transportation, or lost wages for time spent at the clinic (e.g., 10,000 TZS ≈ roundtrip transportation for 3 clinic visits, 22,500 ≈ roundtrip transportation for 6 clinic visits). The intervention period was completed between October 2018-June 2019 (six months after enrollment), with a maximum of 27–60 USD (total) disbursed to each cash transfer participant depending on their randomization assignment. At six-months, the trial found that those in the cash arms of the trial were significantly more likely to be virally suppressed at 6 months [3].

We used purposive sampling to recruit female participants aged 18–49 years who were randomized to either of the cash arms and in a relationship at baseline of the trial. Participants were recruited from HIV clinics where they initiated care from November 2019-February 2020, up to eight months after the intervention period, until theme saturation was reached (no new insights emerged from interviewees) [31].

### Theoretical Framework

This study was informed by the Theory on Gender and Power and its extension to HIV [32], which posits that there are three interrelated social structures which contribute to inequitable gender relations and generate different risk factors for negative health outcomes: (1) sexual division of labor; (2) sexual division of power; and (3) cathexis (social norms and affective attachments). We designed an in-depth interview guide according to the theory structures to assess women’s vulnerability to IPV in the context of HIV and cash transfers. Questions examined women’s employment and income generation, expectations about income and household finances (sexual division of labor), financial decision-making and how cash transfers were spent, partner communication about cash transfers, partner support for cash transfers and receiving HIV care (sexual division of power), relationship dynamics and whether they were altered by employment or receiving cash transfers, and risks and experiences of IPV (defined as physical, sexual, or emotional violence or controlling behaviors) in the presence and absence of women earning money (cathexis). Participants were asked both about general experiences in their community and about their individual experiences with their partners.

### Data Collection

A list of all potentially eligible participants and their upcoming HIV clinic appointment dates was generated from baseline trial data. Potential participants attending clinic visits were approached discreetly in the waiting room by female interviewers to present the qualitative study. Upon conducting a final eligibility screening and receiving informed consent, interviews were conducted in Kiswahili by two female interviewers trained in qualitative and gender-based violence (GBV) research ethics [33, 34]. Interviews took place in private spaces of the clinic, were approximately 45–60 min in duration, and audio-recorded. Following each interview, participants were offered an optional document with GBV resources and local referrals; participants could opt to leave the document with the interviewer to reduce unintended adverse effects if others were to become aware of their study participation.

### Analysis

Interviewers produced field notes in English after each interview. Emergent themes from field notes were reviewed and discussed weekly by the study team; this included dialogue around social and cultural expectations of partnered women in Tanzania, reflection on our individual beliefs about women’s finances and position in the household, and consideration of how our differing beliefs, cultures, and personal experiences may influence the interview process or interpretations of the data.

Audio recordings were transcribed and translated from Kiswahili into English. The English transcripts were coded independently in Dedoose version 8.3.17 [35] by two members of the study team (US qualitative study lead and Tanzanian research manager) using content analysis (categorization and thematic analysis) [36]. The second coder also reviewed the transcripts in Kiswahili to ensure accuracy and quality of translation and to capture any social or cultural nuances that may have been lost in the translation process. A combined inductive-deductive approach was used during the coding process; codes were initially developed based on the interview guide and theory with predefined analytical terms (e.g., partner attitude towards women’s income generation (sexual division of labor); disclosure of cash transfer to partner (sexual division of power); IPV related to cash transfer (cathexis)). Coding was done iteratively, with additional codes developed and codes revised as new concepts emerged from the transcripts. Inter-coder agreement was assessed through comparing codes applied to each transcript and identifying discrepancies. Discrepancies were resolved through an open discussion between the two coders; each coder provided their interpretation of the data, the rationale for the code application, and gathered feedback from the other coder until a consensus was reached.

A matrix of codes was created to identify patterns in the data, and distill and describe key concepts and themes. Views and experiences were summarized overall and tabulated by key socio-demographic characteristics, cash arm, participation in the labor force, reported IPV experiences (ever), and whether IPV was related to the money received from the trial. Findings and interpretations were discussed and validated with the local study team.

### Ethics

Human subjects research approval was obtained from the Institutional Review Boards at the University of California, Berkeley [CPHS 2017-12-10558] and National Institute for Medical Research in Tanzania (NIMR/HQ/R.8a/Vol. IX/2677). The study conforms to the principles embodied in the Declaration of Helsinki.

## Results

Overall, 26 women living with HIV were recruited to participate in the study from November 2019-February 2020, with a 100% participation rate among those approached. One participant disclosed during her interview that she was not partnered during the trial, contrary to baseline data, and was excluded from the analysis. Of the remaining 25 participants, 18 were married (72.0%), fifteen (60.0%) reported experiences of physical, sexual, or emotional violence (e.g., anger/yelling, insults, humiliations, intimidation, threats) or controlling behaviors (e.g., restricted contact with friends or family, required permission to leave the home, anger for talking with other men, suspicion about being unfaithful) in the past 6 months (Table 1). Controlling behaviors (60.0%) and emotional violence (40.0%) were the most frequently reported forms of IPV.

**Table 1:** Demographic characteristics of the study population^a^

	Overall (n = 25)
**Cash arm**	
10,000 TZS (~$4.50 USD)	17 (68.0%)
22,500 TZS (~$11 USD)	8 (32.0%)
**Age**	
Mean ± SD	35.1 ± 7.84
Median (IQR)	37.1 (28.0–40.0)
**Marital status**	
Married	18 (72.0%)
Unmarried with partner	7 (28.0%)
**Attended primary school**	15 (60.0%)
**Employed for wages**	7 (28.0%)
**IPV overall** ^b^	15 (60.0%)
**Controlling behaviors**	15 (60.0%)
**Emotional IPV** ^b^	10 (40.0%)
**Physical or sexual IPV** ^b^	6 (24.0%)
**Sexual IPV** ^b^	4 (16.0%)
**Physical IPV** ^b^	4 (16.0%)

^a^ Data reported at baseline of the trial

^b^ Past 6-month IPV

Participant interviews revealed four main themes: (1) women’s earnings are a source of household tension; (2) small cash transfers have limited influence on relationship dynamics for most women; (3) perceived risks of violence influence disclosure about cash transfers; and (4) partners of cash transfer recipients support access to HIV care. These themes are further described and illustrated in detail below.

### Women’s Earnings as a Source of Household Tension

Most women described money as a common source of household tension and emotional and physical violence in their community. Many reported that men disapprove of women earning an income and characterize such women as “arrogant”.

*“He will get angry and say, ‘this woman is being arrogant because she has money’, so I better beat and injure her so tomorrow she doesn’t go [to work] again. So that situation is just there, it is very few men who can agree that a woman can have money.” − 26 years, married, 10,000 TZS*

Some women shared their direct experiences, explaining that their partners are skeptical of the money they earn and suspect they are getting it from having relationships with other men rather than from their jobs.

*“If he sees me with money maybe he might take it wrongly and think that I got it from men, and he can start to beat you instantly.” − 25 years, partnered, 10,000 TZS*

Most women said they were working small jobs such as selling fruit or farming, or were employed for a wage. However, a few women were not permitted to work, even if they had worked previously. One participant described how she was not allowed to continue working after getting married.

*“Frankly, I was used to getting my own money and my own requirements, not asking anyone for money. Honestly, I feel bad but because I don’t have power, I am a woman and I decided to be married. I had to get used to it, but it took me very long, it took me like four years.” − 37 years, married, 10,000 TZS*

In many cases, women who were working reported that their partners took some of their earnings. Participants had mechanisms to protect their income and avoid conflict, such as hiding their money.

*“I didn’t want to make him mad. I was selling well and I planned to open a business with the money. But at the end of the day, he started taking the money…If he is not around I can sell one jug [of coffee] and get like five thousand [shillings] which I will keep on a phone...without him knowing. When he comes [back] he finds I have put another jug [of coffee] out. I tell him business is slow, because I have my own needs, so I use that trick.” − 25 years, partnered, 10,000 TZS*

### Small Cash Transfers have Limited Influence on Relationship Dynamics for Most Women

In general, women viewed cash transfers differently than money earned from a job, explaining that the amount of the cash transfers was not substantial enough to shift their individual or household financial status. The cash alleviated short-term financial stress and provided temporary food security for some; however, was considered a small amount of money to offset household expenses rather than a dependable supplemental income. Only three participants reported using the cash to invest in their work or businesses. The majority of women reported using the money to purchase household items such as food, school supplies, or other small items for their personal use.

*“I think it helped me with minor issues like transportation or if I had a fever. If you have a headache you can buy panadol [pain relief medication] …you might want fruit and buy it, but you can’t say it has dramatically changed my life.” − 38 years, partnered, 10,000 TZS*

When asked whether the cash transfers led to physical or sexual IPV, none of the women reported that their partners responded with violence after they told them about the money, including those who said they were currently experiencing these forms of IPV. While a small number of women reported that receiving cash improved relations in the short-term, most participants indicated that it was not enough money to lead to any substantial shifts in their relationship dynamic, neither positive nor negative. The cash transfers did not create significant issues nor did they eliminate underlying stressors motivating violence for most women. The money also did not improve women’s financial independence or decision-making power, regardless of the cash amount they received.

*“He supported [cash transfers] because it helped us…when we received it, we were buying food, we ate, and the days went by. So he supported it and didn’t object to anything…he has no problem with it, we are just normal.” – 37 years, married, 10,000 TZS*

However, for some women, receiving cash transfers exacerbated existing tensions with their partners and increased controlling behaviors and emotional violence. For example, some participants were met with anger and hostility after telling their male partners about the cash transfers. Partners expressed doubts about the source of the money, accusing them of taking it from other men, particularly those who were not working or earning an income.

*“He feels so [angry]...he asks, ‘where does she get the money?’. He thinks she is being enticed [seduced].” − 35 years, partnered, 10,000 TZS*

Some women also experienced economic consequences, with partners withholding usual pocket money intended for daily household expenses and transportation fare to the clinic. Their partners expected them to cover these costs with the cash transfer money.

*He was not giving me money to use and when I asked him why he was like, ‘you are given money from your doctors, is it not enough to use?’...since he knew that I was receiving money from the clinic maybe he thought that I would be spending it for my personal spending rather than buying food, so that is why he decided not to give me his own money.” − 23 years, partnered, 22,500 TZS*

Women were asked what they would recommend to others if they were experiencing relationship challenges due to the cash transfers. Advice varied widely and was often based on their employment status, relationship with their partner, and previous experiences managing financial dynamics with men.​ There was not unanimous agreement for how to best manage the cash transfers and whether safeguards could be put in place to better protect women from potential backlash. Some said that they should hide the money from their partners and not tell them to avoid accusations or suspicion.

*“They [women] should keep it a secret, if you are receiving money and you don’t have work, but if you have a business, it is not a problem.” − 46 years, married, 22,500 TZS*

*“If she [a woman] receives the money she should stay quiet because showing it to her husband the money will be snatched and for her, she will get beaten.” − 40 years, married, 10,000 TZS*

Others suggested that women tell their partners directly or bring them to the clinic to learn about the cash transfers and eliminate any potential misunderstanding.

*“I told her [friend] to be open to her husband, or if he doesn’t believe you, come with him so that he can talk with the nurses.” − 42 years, married, 10,000 TZS*

*“You just tell him the truth…if you have taken cash he will see it, if you have taken it through m-pesa [mobile money] then bring him the SMS and he will see that the money was received and withdrawn, because sometimes if you have not told him and he comes to see the SMS it will be a problem. He might say that you have been enticed [seduced], so you have to be open to him, you should not hide it from him.” − 38 years, married, 10,000 TZS*

Women explained that even if they experienced negative consequences with their partners, receiving cash transfers did not lead to any major life changes, both for themselves as individuals and in their relationships. However, many expressed that participating in the study made them feel supported and cared for after learning about their HIV diagnosis and initiating treatment. The incentives were beneficial for their emotional wellbeing and served as a moral boost during a time of deep turmoil.

*“I was happy because it was a very difficult period at that time... I was thinking how would I live while I am already like this [HIV-positive]. I gave up, I even wished to hang myself and die, I didn’t see what value I have here in the world, but I found they [study team] consoled me because when I take it [money] I will buy what I want. I will use it. So, I was comforted in that way.” − 38 years, married, 10,000 TZS*

### Perceived Risks of Violence Influence Disclosure About Cash Transfers

Interviews revealed that women were intentional about whether they chose to tell their partners about the money they received through the trial. Their decision was contingent on several factors, including household finances, financial norms in their relationship, their employment status, perceived safety, concerns of potential backlash, and whether their partner was also participating in the study.

Most disclosed their participation to their partner soon after beginning the study. For many, it was normal and expected to tell their partners when and if they were bringing home money. Others felt that it was safer to tell their partners than to hide it from them. They did not want their partner to learn about their participation through reading their text messages with mobile money transfer receipts or through word of mouth in the community.

*“I made him understand that there is a certain organization that gives us money, so that next time he doesn’t search and find a [text] message and inquire where the money came from when you have not told him. Won’t that cause a fight? Therefore, the first day I make him understand about the issue so he knows it. Then you will not have a fight.” − 37 years, married, 10,000 TZS*

Women often showed the consent form from the study to their partners as a way to prove the source of the money. For some, explaining the study and showing the consent form quelled their partners’ concerns.

*“I told him I was sent money. He asked about who sent me the money, I told him it is from the hospital – there is a group of researchers who have sent me money. ‘How much?’ Ten thousand [shillings]. He got angry because he thought it was sent to me by a man. I showed him the [consent] papers and he read them then he said, ‘ok if they have sent you money it is good, which organization is that?’ I told him it deals with research at the hospital, he said ‘aah cool.’ It was like that.” − 25 years, partnered, 10,000 TZS*

When participants were asked how their partners reacted after they disclosed their participation in the trial, the majority said that they were indifferent – neither happy nor upset.

*He seemed normal, he was not even asking, even when I went alone ... he was just asking if I got the money today and I told him ‘yes, I received it.’” − 40 years, married, 10,000 TZS*

Some participants had partners that were supportive and encouraged them to attend the clinic so they would receive the money, as it relieved some financial pressure within their household.

*“He was supporting it and encouraging me…He just felt good, that day I received that 10,000 TZS we sat together…‘if you are given money, congratulations’, he said.” − 22 years, married, 10,000 TZS*

However, other participants chose not to disclose their participation in the trial due to fear that their partner would stop providing them with pocket money if they knew they were receiving cash from other sources.

*“I felt that he would be depending on me more…that he would be expecting that I would be coming with money, so he would stop leaving money knowing that I would be using the money I bring [from the clinic] to buy household requirements. So, I decided not to tell him.” − 26 years, married, 10,000 TZS*

A few participants also changed their behavior based on their partners’ reactions to the cash transfers. One woman revealed that although she showed the consent form to her partner, he still did not believe that the cash came from the study, so she stopped telling him when she received the subsequent transfers.

*“The first time I received it, I involved my husband. I told him I was sent money on my phone from the clinic. He asked me ‘where is it?’ I showed him. After showing him it became a problem. He said ‘you have been given [the money] by men’, I told him ‘no’ and I showed him the certificate we were given [consent form]. He read it but didn’t believe it, so I decided to let him be, it became my secret.” − 25 years, partnered, 10,000 TZS*

Another participant noted that she stopped telling her partner after he denied her transportation fare for the clinic.

*“I felt uncomfortable telling him...he was refusing to give me extra money for transport fees.” − 40 years, married, 10,000 TZS*

Women fell into two categories with respect to disclosing the cash transfers – those who earned money regularly through small jobs or were employed, and those who did not have an income of their own. Women earning a wage used their previous experience to inform whether and how to tell their partner about the cash transfers. They were familiar with how their partners might react and strategized how to tell them in a way that felt safe and unthreatening, if at all. Generally, participants who were already bringing money into the home had less negative consequences and experienced more positive reactions with regard to their participation. Their partners were used to them earning their own money, thus it was a regular part of their household financial dynamic. In contrast, women who were not earning wages had more issues with their partners and had different challenges to navigate since the cash transfers introduced a new financial dynamic.

### Partners of Cash Transfer Recipients Support Access to HIV Care

Irrespective of any existing tensions within relationships, none of the women who disclosed their HIV diagnosis to their partners reported them interfering with or hindering their access to care. On the contrary, most women described their partners being supportive of them seeking care and said their relationship did not pose any challenges going to the clinic. Many partners would verify their clinic cards and regularly remind them of their upcoming appointment dates, encouraging them to seek treatment.

*“It has reached a point that he is the one who has set an alarm so when it reaches the time for me to take my medicine and I am busy he reminds me.” – 37 years, married, 10,000 TZS*

Notably, women who reported IPV from their partners also described their partners as being supportive of their decision to seek treatment.

*“Even if he has beaten me at night…he looks at my card [and says] today is your visit date, so go. And if I am late, he hires a bicycle so I can be on time. He has never stopped me.” – 38 years, partnered, 10,000 TZS*

Women shared that their partners would often give them money for transportation fare or arrange transport to ensure that they could access treatment. Some also described their partners buying or preparing food to take with their medication.

*“When it comes time to take the medication he reminds me. I told him I should be eating fruits and vegetables. He agreed and used to bring them for me.” − 23 years, partnered, 22,500 TZS*

Overall, participants felt positively about their access to HIV care and did not report any barriers to care pertaining to their relationships.

## Discussion

This qualitative study elicited perspectives from women living with HIV who received up to 6 months of cash transfers through a randomized trial in Shinyanga Region, Tanzania to understand how cash transfers affect relationship safety and IPV. We found that women’s employment is a source of relationship tension and violence in households. However, none of the participants reported physical and sexual violence in relation to cash transfers, including women who were already experiencing these forms of IPV. Cash transfers were viewed differently than income given their small size ($4.5 and $11 USD), and some partners were supportive of women receiving money through the HIV clinic. Although for other women, cash transfers led to emotional violence or controlling behaviors, including anger, accusations that the money came from other men, and withholding of money, particularly those who were not earning a regular wage. Showing the cash transfer trial consent form to partners was used as a strategy by some women to relieve concerns about the source of the money and resolve disputes.

Cash transfers were mainly used for small household expenses and women in both cash arms did not view the amount to be substantial enough to shift the financial dynamic or to tip the balance of power within their relationships. The short duration also limited the extent to which household dynamics were affected in the longer-term, although the transfers did temporarily improve well-being and provide a morale boost at a vulnerable time after their HIV diagnosis. Moreover, women receiving cash transfers did not report partners interfering with their HIV treatment, and were often encouraged and supported to seek care, regardless of their previous or current IPV experiences.

Our data are consistent with previous research in Tanzania demonstrating that for many women, income generation and employment can pose threats to existing gender roles depending on the couple’s financial dynamic [37, 38]. A study in the nearby community of Mwanza found that women in the general population earning a higher income than their partners experienced increased odds of IPV compared to women contributing the same or less than their partners to the household [37]. Taken together, these results suggest that our findings on women’s earnings and relationship tension are unlikely to be unique to women living with HIV and may be applicable to other women living in Tanzania.

However, other studies in Eastern and Southern Africa have also shown that unlike income, small cash transfers for women do not substantially alter intrahousehold relationships [39] and are predominantly used for basic needs and household items [40] similar to what was found in this study. This is in contrast to larger cash transfers provided through social safety net programs which have been shown to increase IPV [15], demonstrating that small incentives designed to subsidize costs of food or transportation may not affect power dynamics in the same ways as a regular wage or larger monetary sums provided in longer-term anti-poverty programs.

These findings indicate that small, short-term cash transfers may be safe for partnered women living with HIV when provided as a behavioral nudge to improve a health behavior, while underscoring that the size of the incentive is likely a central determinant of whether and how women experience aggression and violence related to the transfer. Our results also suggest that providing cash transfers for individuals initiating care may potentially increase support from partners to access ART, however we were not able to compare experiences with ART-initiates who were not receiving cash transfers.

Nevertheless, given the complex relationship between women’s earnings, household financial status, and violence, it remains critical to design and implement economic interventions with caution [41]. Many studies have shown conflicting associations between women’s economic empowerment and IPV depending on the geographical location and socioeconomic context [41, 42]. This study further demonstrates how even with small, short-term cash transfers, some women can be vulnerable to controlling behaviors or emotional violence when provided with money. These behaviors can be more subtle and harder to assess than physical or sexual violence, as they are often less visible and more difficult to measure. Consultations with local female beneficiaries should therefore be prioritized prior to the implementation of cash transfer programs to assess whether additional safeguards or partner communication strategies are needed to protect them from potential harms. For example, the use of duplicate consent forms for women to mitigate the threat of violence is an unexpected and important finding and suggests that documentation on the purpose of cash transfers should be made available for women to share with their partners. The size of the cash transfer and mode of delivery (i.e., mobile, in-person) should also be further explored in future studies to determine how the quantity and mechanism influence women’s safety.

This study builds on the existing evidence base by qualitatively exploring how women living with HIV, a subgroup at increased risk of IPV, are uniquely influenced by cash transfers. While rigorous quantitative studies have investigated the effects of cash transfers on IPV in various contexts, IPV is frequently reported as a binary indicator, limiting our capacity to understand the spectrum of violence that can occur within a relationship. Using the Theory on Gender and Power and its extension to HIV as a guiding framework, we were able to qualitatively examine various, intersecting pathways by which money can alter a woman’s vulnerability to IPV. Including women’s voices allowed us to provide more nuanced insight into the range of harms and benefits women might experience through receiving cash transfers, such as anger, suspicion, and withholding of money, or improved well-being and partner support to receive care. Our results also offer more clarity on how different amounts and sources of money (employment vs. short-term cash transfer intervention) can affect relationships differently and thus why the design and delivery of economic interventions can play an important role in whether women experience violence. In addition, by interviewing women after the intervention period ended, participants had an opportunity to reflect on the ways in which the cash transfers altered their relationships in the longer-term.

However, the 1–8 month delay between the intervention and interview may have led to poor recall of the proximal effects of cash transfers. Other limitations in this study include potential underestimation of IPV in a context where violence is common. Despite efforts to encourage participants to share their experiences, women understandably remained reluctant to discuss violence in their relationships or community. Violence against women is systematically underreported and interviews relied on self-reporting, thus it is possible that some women did experience physical or sexual IPV because of the cash transfers and opted not to disclose during interviews. This study also did not elicit perspectives from male partners of participants in the cash transfer intervention, which may have offered additional insight into the drivers of violence in the context of financial incentives and income generation. Lastly, study recruitment was conducted at HIV clinic sites prior to appointment times, therefore our sample did not include women who missed their scheduled appointment or were disengaged from care.

In conclusion, this study suggests that small, short-term cash transfers do not increase physical or sexual IPV for women living with HIV. However, cash transfers can increase other forms of IPV such as controlling behaviors or emotional violence, thus consultations with beneficiaries should be prioritized while designing incentive programs to protect women from potential risks. Moreover, offering financial incentives as a behavioral nudge may affect women differently than larger amounts aimed at providing household economic relief or empowerment. Additional research is needed to examine how the size and delivery of cash transfers alter relationship dynamics, and how to manage the delicate balance between economic support and power relations within intimate relationships.

## Data Availability

Data provided upon official request.
